# Comparison of ^18^F-FET-PET- and MRI-based target definition for re-irradiation treatment of recurrent diffuse glioma

**DOI:** 10.1016/j.ctro.2025.100931

**Published:** 2025-02-11

**Authors:** Albert Everard, Daniel Versnel, Veerle Ruijters, Nelleke Tolboom, Marielle Philippens, Tom Snijders, Joost Verhoeff, Szabolcs David, Casper Beijst

**Affiliations:** a Department of Radiotherapy, University Medical Center Utrecht, Heidelberglaan 100 3584 CX Utrecht, The Netherlands; b Department of Neurology and Neurosurgery, UMC Utrecht Brain Center, University Medical Center Utrecht, Utrecht, The Netherlands; c Department of Radiology and Nuclear Medicine, University Medical Center Utrecht 3584 CX Utrecht, The Netherlands; d Department of Radiation Oncology, Amsterdam University Medical Center, De Boelelaan 1117 1081 HV Amsterdam, The Netherlands

**Keywords:** FET-PET, Diffuse glioma, Recurrence, Re-irradiation, Tissue composition, Biological tumor volume

## Abstract

**Background and purpose:**

The target area definition for re-irradiation of recurrent glioma typically relies on T1-weighted gadolinium-enhanced MRI (T1Gd), but T1Gd fails to capture glioma infiltration. While T2 FLAIR and CTV margins can address this limitation, their extend in re-irradiation remains debated to minimize toxicity. In contrast, ^18^F-FET-PET imaging can better visualize infiltrative components, improving target definition. This study investigates the role of ^18^F-FET-PET in target definition by analyzing shape differences and gray-white matter tissue composition.

**Material and methods:**

Retrospective data from 36 patients with recurrent glioma were used. These patients underwent ^18^F-FET-PET, on which Biological Tumor Volume (BTV_PET_) was delineated, and T1Gd, on which Gross Tumor Volume (GTV_MRI_) and Clinical tumor volume (CTV_MRI_) were delineated. Lesion volume, shape (Hausdorff distance (HD) and Center of Mass Distance (CoM-D)), tissue composition and re-recurrence volume were compared between the delineations.

**Results:**

BTV_PET_ and the non-overlapping BTV_PET_-GTV_MRI_ volumes were significantly larger than GTV_MRI_ and non-overlapping GTV_MRI_-BTV_PET_ (P < 0.001), but significantly smaller than the CTV_MRI_ (P < 0.001). The median HD and CoM-D were 19 mm and 6 mm. A correlation was found between the white matter (WM) ratio in GTV_MRI_ and WM in non-overlapping BTV_PET_ (slope = 0.36, R^2^ = 0.36;P < 0.001) and a correlation with gray matter (GM) in non-overlapping BTV_PET_ (slope = -0.15, R^2^ = 0.11;P < 0.05). Recurrence after reirradiation was significantly higher within BTV_PET_ than GTV_MRI_ (P < 0.001). The ratio of the GTV_MRI_ which recurred was significantly higher than the non-overlapping BTV_PET_ (P < 0.001).

**Conclusion:**

Our results suggest that ^18^F-FET-PET reveals a more complete extent of glioma’s infiltration, suggesting that the current practice of using isotropic CTV margins around the T1Gd-GTV should be reconsidered in favor of anisotropic CTV based on ^18^F-FET-PET.

## Highlights

• There is a large difference between in shape T1Gd and FET-PET based delineation.
• FET-PET-based radiation plans seem to reflect the glioma infiltration in WM.
• Replacing the CTV-based margin with FET-PET information, reduces treatment volumes.

## Introduction

High-grade diffuse gliomas are among the most common primary brain tumors in adults [1]. Despite advances in surgery, radiotherapy and chemotherapy, recurrence is currently inevitable [2]. In case of tumor regrowth, re-irradiation is among the primary options to prolong overall survival and achieve tumor control [3]. Precise delineation of the Gross Tumor Volume (GTV) is essential for planning re-irradiation treatment, which is particularly challenging in recurrent glioma due to radiation-induced effects that mimic tumor progression on MRI scans [4].

In clinical practice, GTV delineation relies mostly on contrast-enhanced T1 gadolinium (T1Gd) magnetic resonance imaging (MRI) [5]. However, T1Gd enhances areas of the brain where the blood–brain barrier (BBB) is disrupted. Here, it fails to discern radionecrosis from active tumor [6]. Next, T1Gd also fails to capture the non-BBB disrupted parts of the glioma, which includes the tumor components that infiltrate adjacent brain regions. Such infiltration is an integral characteristic of diffuse tumors that ultimately contributes to poor prognosis [7]. To address this limitation of T1Gd, large Clinical Target Volume (CTV) constructed with isotropic margins of 15–20 mm around the GTV are typically employed in newly diagnosed gliomas. [5] This approach, however, often results in collateral damage to healthy or healthy-appearing brain tissue. This issue is of special importance in re-irradiation, as patients have already undergone extensive treatment with significant local toxicity. Consequently, margins should be as tight as possible. There is no clear consensus on CTV margins in glioma re-irradiation, as treatment doses vary widely across countries and institutions [8]. The recent ESTRO/EANO recommendation suggest margins between 3–5 mm, depending on volume and dose prescription [9]. Amino acid PET tracers, such as O-(2-[^18^F]-fluoroethyl)-L-tyrosine (^18^F-FET), can penetrate the intact BBB, providing better visualization of areas of glioma infiltration and thus improving tumor delineation by contouring a Biological Tumor Volume (BTV) [10]. Biopsy studies support ^18^F-FET-PET’s high specificity (93 %) compared to MRI (54 %) in tumor localization [11], [12]. ^18^F-FET-PET has shown value in improving presurgical identification of glioma tissue over conventional MRI [13]. Recurrence studies also show that ^18^F-FET-PET positive volumes align with patterns of relapse post-treatment [14], underscoring its utility in glioma localization.

^18^F-FET-PET has been shown to lead to substantial alterations in defining GTV and CTV for both newly diagnosed and recurrent glioma, as established in several studies [15], [16], [17], [18]. These works have revealed a significant discrepancy in tumor volume measurement between ^18^F-FET-PET and MRI. Recent consensus publications suggest a potential role for ^18^F-FET-PET imaging in radiation planning [19] and pave the way for clinical trials investigating its use in target delineation of primary gliomas, such as the FIG trial [20]. However, there is currently no clear agreement within the radiation oncology community regarding its application in target definition, as shown in the recent pictorial guide indicating potential pitfalls [21]. This is particularly true for re-irradiation treatments, where the balance between tumor control and treatment related toxicity is especially delicate. The GLIAA trial, which aimed to provide a definitive answer on whether ^18^F-FET-PET as target definition for re-irradiation results in better clinical outcome, yielded negative results [22]. This highlights the need to rethink how ^18^F-FET-PET should be implemented in re-irradiation target definition.

For this purpose, an in-depth understanding of recurrent glioma target definition by ^18^F-FET-PET is essential, including differences in tumor shape and white and gray matter tissue composition between ^18^F-FET-PET and MRI, areas that remain unexplored in previous studies. Therefore, the aim of this study is to investigate the role of ^18^F-FET-PET in revealing a more complete extent of glioma’s infiltration by assessing gray-white matter tissue composition, difference in shape and volume, and subsequent re-recurrence patterns of T1Gd- and ^18^F-FET-PET-based delineations.

## Materials and methods

This is a mono-center retrospective cohort study, performed at the University Medical Center Utrecht. All original data were collected as part of routine clinical care. The study was approved by the ethics committee (23U-0098).

### Patient inclusion

Forty-six patients that underwent ^18^F-FET PET/CT for reirradiation treatment of high-grade adult diffuse glioma (WHO grade 2,3 and 4) between 2012 and 2023 were included. The patients were scanned on Biograph mCT PET/CT (Siemens Healthineers) using a 30 min static scan protocol and a reconstruction method incorporating point spread function modelling and time-of-flight. The MRI scans were acquired on Ingenia 3 T Philips MRI with a T1 weighted Spoiled gradient echo after gadolinium administration.

Inclusion criteria comprised of all patients who underwent ^18^F-FET PET/CT for reirradiation-planning at UMC Utrecht, including those who did not ultimately receive reirradiation. Patient were clinically eligible for radiation-planning with ^18^F-FET-PET when they had larger lesions; lesions with poorly defined borders on MRI; and/or lesions with a presumed component of radionecrosis. Patients underwent follow-up MRIs one month post-reirradiation, followed by subsequent scans every three months. The prescribed dose was typically 10 x 3.5 Gy (EQD2: 48 Gy), but could vary based on specifics of each cases. Patient characteristics are summarized in Table 1.

**Table 1:** Patient Characteristics.

*Characteristics*	n
N patients	36
Age, y, median (range)	50, (30-70)

Sex:	
Male	23
Female	13

WHO Classification	
Astrocytoma IDH-mutant	16
*Grade*	
2	3
3	6
4	7
Oligodendroglioma 1p/19q-	4

codeletion	
*Grade*	
2	2
3	3
Glioblastoma IDH-wildtype	6
*Grade*	
4	6
Astrocytoma NOS	3
(suspected IDH-mutant)	
*Grade*	
3	3
Glioblastoma NOS	4
(suspected IDH-wildtype)	
*Grade*	
4	4

ReRT Dose	
10 × 3.5 Gy	13
28 × 1.8 Gy	8
28 × 1.8 Gy + Boost 2.1 Gy	6
30 × 2 Gy	2
8 × 3.5 Gy	1
SRT 3 × 7 Gy	1
No reRT	5

### Delineations

**Fig. 1:**
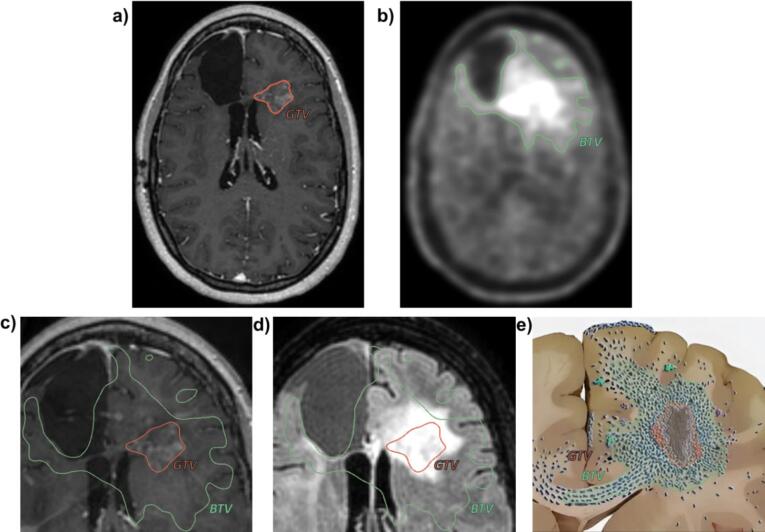
An example of the GTV and BTV delineations. a) A T1Gd scan overlaid with corresponding GTV contouring all the enhanced part on the image. b) A 18F-FET-PET scan with the corresponding BTV constructed with a cut-off value of 1.6 TBR. c) The corresponding GTV and BTV overlaid on the T1Gd. d) The corresponding GTV and BTV overlaid on the FLAIR. e) An infographic illustrating which parts of the glioma are visualized by T1Gd and which by FET-PET. .

Adapted from image of A. Claes et al. [38]

For this study, new GTV_MRI,_ BTV_PET_ and CTV_MRI_ were delineated with our in-house developed clinical RT-delineation software, Volume-tool [23]. The GTV_MRI_ was delineated on T1Gd by contouring the contrast-enhanced regions. The CTV_MRI_ was constructed by enlarging the GTV_MRI_ with 1 cm margins taking anatomical boundaries into account, which is protocol for re-irradiation of gliomas in the UMC Utrecht. The BTV_PET_ was semi-automatically delineated with use of a Tumor-to-Background Ratio of 1.6 as cutoff value, based on recommended values from biopsy studies [12]. The mean Standard Uptake Value of the background was calculated to determine Tumor-to-Background Ratio. This was done by delineating a region-of-interest in the healthy contralateral hemisphere in which the Standard Uptake Value mean was determined [24]. Areas that were PET-avid but not part of the tumor were not considered. An example of delineations is shown in Fig. 1.

### Recurrence analyses

Recurrence post-re-irradiation was defined on the first follow-up T1Gd scan showing new enhancement with an ultimate diagnosis of a new recurrence; lesions that were ultimately classified as completely consisting of radionecrosis were not included for this analysis. Re-recurrence GTV_MRI_ (rGTV_MRI_) was visualized by delineating all enhancing parts on the T1Gd MRI scan with use of the Smartbrush tool of Brainlab Elements (Brainlab AG). First the ratios of rGTV_MRI_ overlapping with the BTV_PET_ and GTV_MRI_ were calculated. Secondly, the percentage of the delineations which recurred after radiation was calculated. Lastly the volume which received 95 % of the prescribed dose (V95) was calculated for the rGTV_MRI_ to determine whether the newly recurred glioma volume received 95 % of the prescribed re-irradiation dose, which can indicate if the dose constrains were sufficient for an adequate tumor control.

### Registration

To facilitate the comparison of BTV_PET_, GTV_MRI_, and rGTV_MRI_ delineations, all images were co-registered. Rigid registration of CT, acquired from the PET/CT scan, and follow-up T1Gd scans to the treatment T1Gd scan was conducted using Volume-tool for the CT, and SPM12 (https://www.fil.ion.ucl.ac.uk/spm/software/spm12/) for the follow-up scan. Subsequently, the transformation was applied to the delineations and PET scan to align with the treatment T1Gd scan.For GM and WM tissue segmentation, a non-linear registration to MNI (Montreal Neurological Institute) brain template was performed. This involved registering the T1Gd to an MNI template using SPM12. The transformation matrix derived from this registration was then applied to all other scans and delineations that were previously aligned with the T1Gd, thus bringing all scans and delineations into same coordinate system as the MNI template. The scans and delineations were visually inspected after registration, to insure proper registration.

### Volume and shape analyses

Volumetric comparison of GTV_MRI_, BTV_PET_ and CTV_MRI_ was conducted for each patient by calculating absolute volume. The non-overlapping and overlapping volumes were calculated between the GTV_MRI_ and BTV_PET_. Similarly this was calculated between CTV_MRI_ and BTV_PET_. the Shape similarity between BTV_PET_ and GTV_MRI_ was quantified using Hausdorff distance (HD) calculate with MONAI and center of mass distance (CoM-D). The HD represents the maximum contour distance between delineations and CoM-D indicates directional skewness of the delineations.

### Tissue composition analyses

Tissue composition analyses were conducted to estimate the ratios of white matter (WM) and gray matter (GM) within the BTV_PET_, GTV_MRI_, and non-overlapping BTV_PET_. WM and GM ratios were calculated using MNI probability templates. Delineation masks in MNI space were multiplied in a voxel-wise manner with these tissue probability maps to generate WM and GM probability masks for each delineation. The volumes of these probability maps were then divided by the absolute volumes of the delineations to obtain the WM and GM ratios.

### Statistical analyses

Wilcoxon test was performed with Graphpad (version 10.0.0 for Windows, GraphPad Software, Boston, Massachusetts USA, https://www.graphpad.com) to statistically compare differences in volume and tissue composition of delineations. Linear regression was conducted with use of R-studio to explore correlations between calculated metrics (HD, volume) and patient clinical characteristics (Table 1), as the shape and volume of GTV_MRI_ and BTV_PET_ may be influenced by these clinical characteristics. This was done in two stage. First with a univariant linear regression with WHO classification as determined and GTV_MRI_ volume, BTV_PET_ volume and HD as independent variables. Secondly, a multi variant linear regression with age added as determined, since WHO classification is age dependent.

## Results

### Patient characteristics

Of the 46 patients eligible for the study, 36 patients (n = 23 male, n = 13 female) with a median age of 50 years (range: 30–70 years) met the inclusion criteria. Reasons for exclusion were: no T1Gd MRI performed within two weeks of the ^18^F-FET-PET (n = 1), insufficient uptake of the tumor (<1 cc) on ^18^F-FET-PET (n = 3) or T1Gd (n = 8). The WHO 5th classification included: Astrocytoma (IDH-mutant) n = 16 with n = 12 radionecrosis, Oligodendroglioma (IDH-mutant with 1p/19q-codeletion) n = 4 with n = 2 radionecrosis and Glioblastoma (IDH-wildtype) n = 6 with n = 3 radionecrosis. The IDH-status of seven patient was unknown and are indicated as not otherwise specified (NOS) with their suspected IDH-status based on histomorphology and clinical presentation. At a median of 54 (IQR: 34, range: 204) months after the first RT the patient received re-irradiation, the radiation fractionation schedules are indicated in Table 1. Irradiation was performed using Elekta Versa HD and Elekta Agility. The key characteristics are summarized in Table 1.

### Volume and shape analyses

**Fig. 2:**
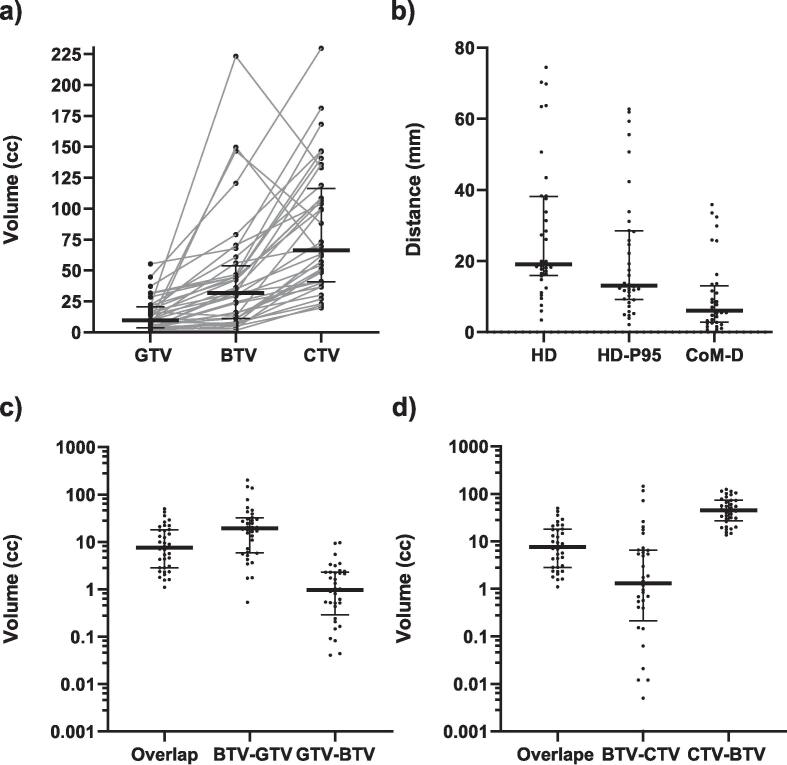
Volume and shape metrics. a) The median and individual absolute volumes of the Gross Tumor volume (GTV), Biological Tumor Volume (BTV) and clinical target volume (CTV) in cc. The lines between the dots indicate the paired delineations per patient. d) The median and individual Hausdorff distance (HD), Hausdorff distance 95 percentile (HD-P95) and center of mass distance (CoM-D) between the GTV and BTV. c) The median and individual volumes of the overlap of the biological tumor volume and gross tumor volume, the non-overlap of the biological tumor volume (BTV – GTV) and the non-overlap of the gross tumor volume (GTV – BTV) in cc with a logarithmic scale. d) The median and individual volumes of the overlap of the biological tumor volume and clinical tumor volume, the non-overlap of the biological tumor volume (BTV – CTV) and the non-overlap of the gross tumor volume (GTV – CTV) in cc with a logarithmic scale.

The median volume of the GTV_MRI_, consisting of 10 (IQR: 4–21) cc, is significantly smaller than the median volume of the BTV_PET_, which is 32 (IQR: 11–54) cc (P < 0.001). The median volume of the CTV_MRI_, consisting of 66 (IQR: 20–230) cc, is significantly larger than the median volumes of the BTV_PET_ (P < 0.001) (Fig. 2a). The median volume of the overlap of the BTV_PET_ and GTV_MRI_ is 8 (IQR:3–18) cc. The median volumes of the non-overlapping parts are 19 (IQR: 6–32) cc for the non-overlapping part of the BTV_PET_ (BTV_PET_ – GTV_MRI_) and 1 (IQR:0–2) cc for the non-overlapping part of the GTV_MRI_ (GTV_MRI_ – BTV_PET_) (Fig. 2c). for the CTV the overlapping volume with the BTV is 8 (IQR: 3–18) cc. The medians were 1 (IQR:0–7) cc for the non-overlapping part of the BTV_PET_ (BTV_PET_ – CTV_MRI_) and 45 (IQR:27–73) cc for the non-overlapping part of the CTV_MRI_ (CTV_MRI_ − BTV_PET_)(Fig. 2d). The shape metrics consisted of a median of 19 mm for the HD, 13 mm for the 95th percentile HD (HD-P95) and 6 mm for the CoM-D (Fig. 2b).

### Tissue composition

**Fig. 3:**
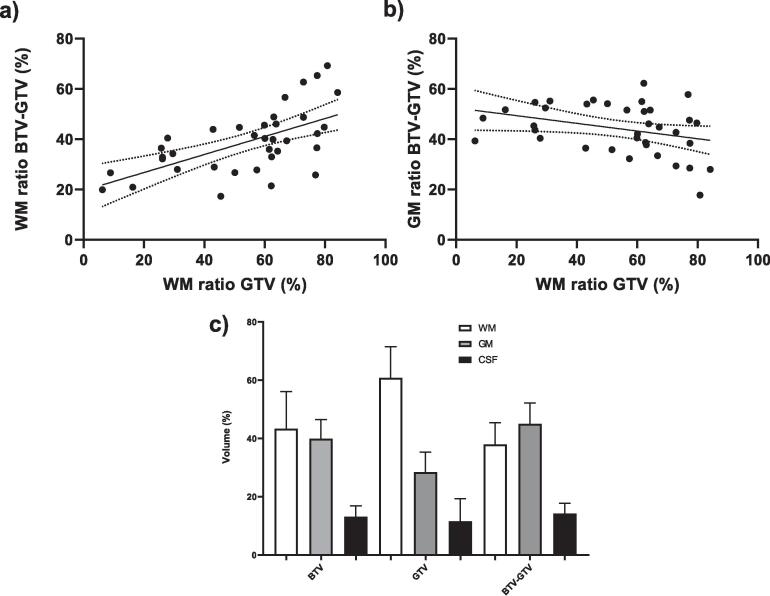
Graphs of the ratio WM and GM inside the delineations. a) The proportion of WM of the total volume of the non-overlapping BTV (BTV – GTV) plotted against the ratio of WM of the total volume of GTV in percentage with a linear regression fit. b) The percentage of GM of the total volume of the non-overlapping BTV (BTV – GTV) plotted against the proportion of WM of the total volume of GTV in percentage with a linear regression fit. c) The median proportion of the tissue types of the total volume of the BTV, GTV and non-overlapping (BTV – GTV).

The median proportion WM (61 %) is significantly larger than the proportion of GM (28 %) for the GTV_MRI_ (P < 0.001). For the BTV_PET_ and non-overlapping BTV_PET_ (BTV_PET_ – GTV_MRI_), there is no significant difference between WM and GM ratios (Fig. 3c). However, there is a positive correlation between the WM ratio of the GTV and the WM ratio of the BTV_PET_ – GTV_MRI_ with a slope of 0.36 and R^2^ of 0.37 (P < 0.001) (Fig. 3a). Moreover, there is a negative correlation between the WM ratio of the GTV_MRI_ and the GM ratio of the BTV_PET_ – GTV_MRI_ with a slope of −0.15 and a R^2^ of 0.11 (P = 0.048) (Fig. 3b).

### Recurrence analyses

**Fig. 4:**
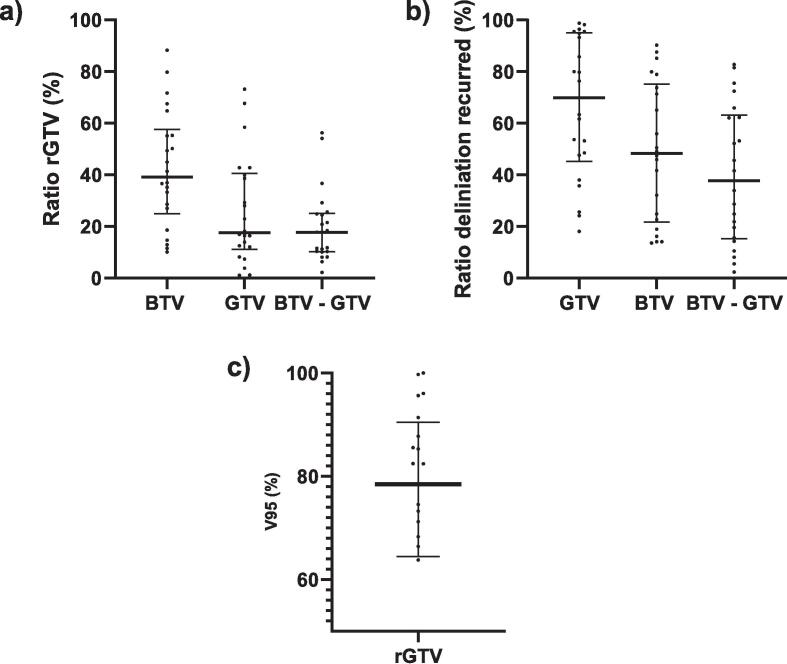
Shows the graphs of the recurrence analyses. a) The percentage of the overlap between the BTV, GTV and non-overlapping BTV and the rGTV and the absolute volume of the rGTV per patient and the median. b) The percentage of the overlap between the BTV, GTV and non-overlapping BTV and the rGTV and the absolute volume of the delineations per patient and median. c) The median and individual percentage of the V95 from the rGTV.

Twenty-two of the 36 patients exhibited tumor progression. In 20 of the 22 patient the overlap with the rGTV_MRI_ was greatest for the BTV_PET_. This is also indicate by the median of the overlap with the rGTV_MRI_, which is significantly larger for the BTV_PET_ (39 %) than for the GTV_MRI_ (18 %) and non-overlapping BTV_PET_ (18 %) (P < 0.001) (Fig. 4a). However, the percentage of new recurrence (rGTV_MRI_) within the GTV_MRI_, BTV_PET_ and non-overlapping BTV_PET_ is the highest for the GTV_mri_ in 21 of 22 patient with a median of 70 %, significantly higher than the median of the BTV_PET_ (48 %) and non-overlapping BTV_PET_ (38 %) (P < 0.001) (Fig. 4b). The median V95 in percentage of the rGTV_MRI_ consisted of 79 % (Fig. 4c).

### Correlation of clinical values

Table 2 shows the results of the linear regression models, where the NOS cased were added to their suspected WHO classification. A correlation was found between the BTV_PET_ volume and WHO classification. This indicates that the BTV_PET_ volume of the astrocytoma, IDH-mutant (68 cc, P < 0.001) is significantly larger than the BTV_PET_ volume of the glioblastoma, IDH-wildtype (28 cc, p = 0.039). A similar correlation was found for the HD, where the HD for the astrocytoma, IDH-mutant (36 mm, P < 0.001) is significantly larger than the glioblastoma wild-type (20 mm, P = 0.026) (Table 2). The multivariant linear regression models with age added as determinant showed the correlation remains significant for HD but not for the BTV_PET_ volume.

**Table 2:** Overview the results of the univariant linear regression model with BTVPET volume, GTVMRI volume and HD as independent variable and as determinant WHO classification and multivariant linear regression model with age added as determinate, where the mean of the WHO classification were calculate at age 30y. Significant findings (p < 0.05) are given in bold.*: Change per age step.

*Univariant*	**Volume BTV**		**Volume GTV**		**HD**	
***WHO Classification***	*Estimates*	*P-value*	*Estimates*	*P-value*	*Estimates*	*P-value*
Astrocytoma, IDH-mutant (Baseline)	**62.84**	**<0.001**	**16.27**	**<0.001**	**35.75**	**<0.001**
Glioblastoma, IDH-wildtype	**26.20**	**0.0301**	12,85	0.476	**20,40**	**0.0259**
Oligodendroglioma, 1p/19q-codeletion	16.60	0.0702	7.19	0.220	15.87	0.0565

*Multivariant* ***WHO + Age***						

Astrocytoma, IDH-mutant (Baseline)	**80.05**	**<0.001**	**16.87**	**< 0.001**	**34.79**	**<0.001**
Glioblastoma, IDH-wild type	61,26	0.313	14.06	0.627	**18.47**	**0.0485**
Oligodendroglioma, 1p/19q-codeletion	51,53	0.283	8.40	0.299	13.92	0.0696
Age (Slope)*	−1.25	0.097	−0.17	0.894	−0.07	0.847
*Observations*	36		36		36	

## Discussion

In this study, we compared the target definition based on T1Gd and ^18^F-FET-PET to provide insight into the potential of ^18^F-FET-PET for planning of re-irradiation treatment of progressive diffuse gliomas.

Our results revealed a large discrepancy in volume between the ^18^F-FET-PET and T1Gd-based delineations. This difference was caused by the non-overlapping part of the BTV_PET_, which most likely represent tumor infiltration [25], suggesting that a part of the infiltrated tumor is missed when only using T1Gd. These findings align with previous studies reporting a larger BTV_PET_ volume compared to GTV_MRI_ [26], [27], [10]. The ability of ^18^F-FET-PET to visualize a more complete tumor infiltration suggests that the non-overlapping GTV indicates treatment-associated effects including pseudo-progression and radionecrosis [6].

Shape differences of the target volumes based on ^18^F-FET-PET and T1Gd have been underexplored. In this work, the shape difference was captured by calculating the HD and the CoM-D, which are relatively large. Indicating a large contour distance and asymmetry between the BTV_PET_ and GTV_MRI_ caused by a difference in shape. The median HD of 19 mm is larger than the 1 cm CTV margins used in this study and the recommended CTV margins of 3–5 mm [9] showing that the margins are insufficient for treating the entire glioma.

However, the results show that the isotropic constructed CTV_MRI_ exhibited a larger volume than BTV_PET_, suggesting an overtreatment of the tumor volume with current treatment. This issue may be resolved by using the ^18^F-FET-PET-based BTV to define the CTV. As a consequence, the total treatment volume can be reduced, but the infiltrative part of the glioma may still be treated, which can result in a net more effectively therapy. Leading to a reduction of radiation toxicity with the same stable, or improved, tumor control. This may not apply when using smaller margins, as recommended by the ESTRO/EANO guidelines. However, due to the shape differences between GTV and BTV, a portion of glioma infiltration may be overlooked, potentially compromising the effectiveness of the treatment.

One cause of the shape difference could be the ability of ^18^F-FET-PET to capture infiltration along the white matter tracts. The correlation between the WM ratio of the GTV_MRI_ and ratio WM and GM of the non-overlapping BTV_PET_ indicates that when the GTV_MRI_ core is located in the WM, the infiltration indicated by the ^18^F-FET-PET also has a preference for WM, highlighting the infiltration along the WM tracts, a known biological property of glioma [28]. This supports our hypothesis that ^18^F-FET-PET is superior over T1Gd in capturing more complete infiltration of diffuse glioma, improving radiation treatment of diffuse glioma. For example, the tissue composition information of the ^18^F-FET-PET could be used to constructed anisotropic margins along the WM tracts [29]. Anisotropic margins have been suggested as a solution for overcoming the non-specific nature of the CTV margins. However, constructing anisotropic margins is challenging, hindering clinical adaptation. Currently, the construction of the anisotropic margins is mostly based on DTI atlases [30], even though DTI cannot estimate crossing fibers and lacks tumor information. ^18^F-FET-PET could help better defining these margins, since our results support its ability to visualize tissue-specific growth patterns.

The ability of ^18^F-FET-PET to visualize growth patterns is also underlined by the re-recurrence analysis, suggesting that the BTV_PET_ overlaps the recurrence more than the GTV_MRI_, in line with recent findings [14], [31]. Furthermore, a large part of the GTV_MRI_ volume recurred after re-irradiation treatment. This may suggest that GTV_MRI_, which is determined on the T1Gd enhancing leakage due to BBB disruption, [32] consists of the most aggressive and radioresistant tumor cells. The results also showed that a large part of the recurrence volume received 95 % of the prescribed dose. Taking into account that the tumor has grown, this result suggests that the dose is insufficient to achieve total tumor control, which is also reported in previous studies [14], [33].

IDH-mutant astrocytoma showed significantly larger BTV_PET_ volume and HD compared to other types. Astrocytoma initially as low grade gliomas [34]. Therefore, astrocytomas are less likely to induce BBB disruption at the start of the indication, which leads to leakage visible on T1Gd. As a consequence the astrocytomas might grow without leading to clinical consequent, resulting in a large BTV_PET_ comparted to GTV_mri_.

Overall, the results suggest that ^18^F-FET-PET reveals a more accurate extent of the glioma, especially in gliomas with large differences between T1Gd and ^18^F-FET-PET-based delineations, such as IDH-mutant astrocytomas. Including ^18^F-FET-PET based delineations in the radiation planning may help visualize and treat the actual tumor infiltration, potentially reducing or replacing traditionally isotropic CTV margins leading to a smaller overall treatment volume [17], [35]. In turn, this could lead to reduced radiation toxicity with similar treatment outcome. Despite this improvement, it is unlikely that better tumor control can be achieved, as a large part of the recurrence volume did receive 95 % of the prescribed dose. This result may also help explain the negative outcomes of the GLIAA trail, which compared ^18^F-FET-PET based target definition with standard T1Gd for re-irradiation to asses improvements in progression free survival. However, standard re-irradiation schemes and contouring methods were used in GLIAA, which are likely insufficient for improved tumor control. However, realizing that the current delivered dose is primarily limited by the dose to the healthy surrounding brain tissue in an effort to mitigate radiation toxicity, better tumor definitions with ^18^F-FET-PET-based CTV also lead to future dose escalation opportunities. For example, a boost can be given based on T1gd GTV or ^18^F-FET-PET hotspot, of which the feasibility has been tested, [36], [37] potentially leading to better tumor control.

Our study has some limitations, mostly due to its retrospective nature, which can lead to a selection bias in the patient cohort. In fact, ^18^F-FET-PET scans were not randomly allocated to diffuse glioma reirradiation patients but were based on subjective clinical factors. Furthermore, prior knowledge of the BTV_PET_ of the treating physicians could have influenced the GTV_MRI_ delineation process, although the BTV_PET_ was hidden while delineating the GTV_MRI_. Another limitation was the incomplete information on molecular markers for some patients, reflecting the histopathological standards of their treatment eras. Although this limits the ability to perform detailed correlations between molecular diagnoses and PET-data, it was still possible to provide a plausible classification for all patients. Lastly, the patient population is relatively small and heterogeneous regarding glioma type and dose prescription. Nevertheless, this study provides a better understanding of the role of ^18^F-FET-PET in revealing glioma infiltration, especially in relation to the novel aspect of gray-white matter tissue composition.

In conclusion, our results suggest that ^18^F-FET-PET reveals a more complete extent of growth of a recurrent glioma, in particular the infiltration along white matter tracts. These findings suggest that the current practice of using isotropic CTV margins around the T1Gd-GTV should be reconsidered in favor of anisotropic CTV based on ^18^F-FET-PET. This paradigm can lead to a reduced treatment volume and treatment of true tumor progression, resulting in decreased toxicity. To investigate the clinical feasibility of CTV ^18^F-FET-PET based delineations, further prospective clinical research is warranted. In which the effect of CTV ^18^F-FET-PET based delineations on relevant endpoints including overall survival, progression-free survival and health-related quality of life need to be investigated.

## Authorship

Conception and design: A.J.E, C.B, S.D., J.J.C.V.; Acquisition of data: D.C.A.V, V.J.R, S.D.; Data analyses: A.J.E.; Interpretation of the results: C.B, S.D., J.J.C.V., N.T., T.J.S., M.E.P.P.; Writing, review, and/or revision of the manuscript: A.J.E., C.B, S.D., J.J.C.V., N.T., T.J.S., M.E.P.P. Supervision C.B, S.D.

## Declaration of generative AI in scientific writing

During the preparation of this work the author(s) used open ai chatgpt in order to review the text on grammar and spelling. After using this tool/service, the author(s) reviewed and edited the content as needed and take(s) full responsibility for the content of the publication

## Funding and finical discloser

The Koningin Wilhelmina Fonds (KWF) kankerbestrijding (14848 to C.B). The funders had no role in study design, data collection and analysis, decision to publish, or preparation of the manuscript.

## Declaration of competing interest

The authors declare that they have no known competing financial interests or personal relationships that could have appeared to influence the work reported in this paper.
